# Theoretical Study on the Thermal Decomposition Mechanism of Fe(EDTA)^−^ and Fe(EDTMP)^−^

**DOI:** 10.3390/molecules29184362

**Published:** 2024-09-13

**Authors:** Kai Zhang, Zhan Wang, Shuying Ma, Chen Wu, Xiaoyang Zhao

**Affiliations:** 1 School of Geomatic and Environmental Engineering, Henan Polytechnic Institute, Nanyang 473000, China; 2 Department of Physics, School of Science, Harbin University of Science and Technology, Harbin 150080, China

**Keywords:** Fe(EDTA)^−^, Fe(EDTMP)^−^, thermal decomposition, AIMD

## Abstract

The decomposition mechanisms of Fe(EDTA)^−^ and Fe(EDTMP)^−^ complexes, widely used in various industrial applications, were investigated through a theoretical approach. Despite their structural similarities, the phosphonic acid and carboxylic acid groups in these complexes lead to vastly different decomposition behaviors. Fe(EDTA)^−^, stabilized by delocalized π bonds in carboxylic acid groups, exhibited higher stability than that of Fe(EDTMP)^−^, which has only σ bonds in phosphonic acid groups. Interaction Region Indicator (IRI) analysis revealed that the steric hindrance of Fe(EDTMP)^−^ was stronger than that of Fe(EDTA)^−^. Ab initio molecular dynamics simulations revealed that Fe(EDTMP)^−^ undergoes rapid decomposition due to the ease of breaking P-C bonds and the repulsion between phosphonic acid groups. In contrast, Fe(EDTA)^−^ decomposes more slowly. These findings suggest the incorporation of phosphonic acid groups for easier degradation pathways when designing organic acid molecules. Understanding these mechanisms provides a basis for developing strategies for wastewater treatment in industrial settings.

## 1. Introduction

Ethylenediaminetetraacetic acid (EDTA), as a chelating cleaning agent, possesses several advantages, such as safety, efficiency, and a short working period when used in chemical cleaning [1,2]. EDTA exhibits strong chelating properties with metal ions in scale, effectively removing scales, such as calcium scale [3], calcium carbonate scale [4], and copper scale [5], with minimal corrosion to the metal substrate. Additionally, EDTA, as a widely used chelating agent, plays a significant role in detergents [6], membrane cleaning [7], pharmaceuticals [8], and other fields. However, the long lifespan of EDTA poses serious environmental risks, as it exhibits high persistence in sewage treatment plants and natural water bodies, being classified as one of the major organic pollutants in water [9]. EDTA can also desorb heavy metals bound to sediment and prevent their precipitation, thereby increasing their circulation in water. However, these metal complexes do not have the same bioavailability as free metal ions, and these forms of chelates can lead to the long-term presence of toxic heavy metals in aquatic systems. This affects the balance of essential and non-essential metals in natural water bodies and aquatic organisms, possibly leading to long-term effects [10]. Therefore, efforts should be made to minimize the release of chelates into natural bodies of water.

EDTA degrades slowly in the environment. Kari et al. [11] found that neither biological nor chemical processes significantly eliminated EDTA during sewage treatment, while the phosphorus substitute, nitrilotriacetic acid (NTA), was effectively degraded. Nörtemann et al. [12] found that conventional biological and physicochemical methods used for wastewater treatment and drinking water purification cannot degrade EDTA, though it can be degraded by specific bacterial cultures. Satroutdinov et al. [13] studied the degradation of metal–EDTA complexes by bacterial strain DSM 9103. Pirkanniemi et al. [14] used the Fenton process to degrade EDTA in pulp and paper mill processes, as well as wastewater. Noradoun et al. [15] explored a new method for the degradation of EDTA using zero-valent iron, air, and water to activate oxygen at room temperature. Zhao et al. [16] irradiated wastewater containing Cu(II)-EDTA by ultraviolet irradiation to achieve simultaneous reduction of Cu(II)-EDTA and degradation of EDTA. Szilágyi et al. [17] investigated the thermal stability of the solid monomeric Na[FeEDTA(H_2_O)]·2H_2_O complex using simultaneous TG/DTA and EGA-MS, complemented by Mössbauer spectroscopy. Biegun et al. [18] researched the thermal decomposition characterization and kinetics of Cu, Fe, Mn, and Zn chelates of EDTA in the atmosphere, revealing their decomposition complexity and stability order. Gabelica et al. [19] studied the thermal degradation of iron chelate complexes adsorbed in mesoporous silica and alumina.

Ethylenediamine Tetramethylenephosphonic acid (EDTMP) is an organophosphonic acid with chelating and corrosion inhibiting properties, and it is a phosphonic acid analog of EDTA. EDTMP is typically present in the form of disodium salt, and it exhibits good solubility in water. EDTMP is used as a scale and corrosion inhibitor in water treatment, and it possesses good chemical stability [20,21]. The degradation of EDTMP and its chelates was researched. Matthus et al. [22] studied the photodegradation reaction of Fe(EDTMP). A 254 and 365 nm monochromatic light was used to irradiate a 10^−4^ M Fe(EDTMP) aqueous solution, and the degradation process was monitored by analyzing the formed orthophosphate. Kuhn et al. [23] investigated the non-biological degradation pathway of EDTMP under simulated UV irradiation. EDTMP was degraded within 30 min, with the primary degradation product being iminodimethylene phosphonic acid.

However, the above studies are macroscopic observations and do not investigate the decomposition process of Fe(EDTA)^−^ or Fe(EDTMP)^−^ complexes at the molecular level. This study employs density functional theory (DFT) and ab initio molecular dynamics (AIMD) theory to study the thermal decomposition reactions of Fe(EDTA)^−^ and Fe(EDTMP)^−^ complexes, focusing on the steps of thermal decomposition and providing possible decomposition pathways. This may serve as a reference for their degradation processes in the natural environment and lay the foundation for the design of similar organic acid molecules.

## 2. Calculation Methods and Details

The molecular structures of Fe(EDTA)^−^ and Fe(EDTMP)^−^ complexes were built using GaussView6 and then optimized using Gaussian16 software [24]. DFT calculations were performed at the B3LYP level of theory with the DEF2-TZVP basis set, employing the Solvation Model Based on Density (SMD) implicit solvent model, with water as the solvent. Frequency calculations were carried out to confirm that the obtained structures have no imaginary frequencies, indicating stable structures on the potential energy surface.

These optimized structures were used as the initial configurations for ab initio molecular dynamics (AIMD) simulations. The complexes were placed in a cubic box with a side length of 30 Å and simulated for decomposition reactions under gas-phase conditions using the CP2K/Quickstep program [25,26]. The Quickstep module employs a mixed Gaussian plane wave scheme, where the atomic cores are represented by Goedecker–Teter–Hutter (GTH) pseudopotentials [27], and valence electrons are described using double-ζ polarized (DZVP) basis sets [28]. The Perdew–Burke–Ernzerhof (PBE) exchange-correlation functional [29] was used to describe exchange-correlation interactions, and D3 empirical dispersion corrections [30] were applied to account for van der Waals (vdW) interactions. The temperature was maintained at the desired value of 3000K using a Nośe–Hoover thermostat under the canonical ensemble (NVT) condition to accelerate the decomposition process [31,32,33]. The plane wave cutoff was set to 400 Ry. The simulation time was set to 5000 fs for Fe(EDTA)^−^ and 800 fs for Fe(EDTMP)^−^, with a time step of 0.5 fs.

Visualization of molecular structures and Interaction Region Indicator (IRI) analysis was performed using the VMD program [34], while the AIMD trajectory, bond formation, and bond breakage was visualized using the Jmol program [35], with default settings for the thresholds of bond formation and bond breakage.

## 3. Results and Discussion

### 3.1. Molecular Structure and Steric Hindrance of Complexes

O2 and O3 are referred to as planar O, while O4 and O5 are referred to as axial O. It can be observed from Table 1 that the Fe-O bonds in the planar positions of both complexes are slightly longer than those in the axial positions, which may be attributed to the spatial arrangement of the molecules. The Fe-N bonds are longer than the Fe-O bonds, implying the weaker electronegativity of N compared to O and its weaker coordination ability. A comparison of bond lengths between the two complexes shows that the corresponding Fe-O or Fe-N bonds in the octahedral coordination of Fe(EDTA)^−^ are slightly shorter than those in Fe(EDTMP)^−^, indicating a tighter binding of EDTA to the Fe^3+^ ion than that of EDTMP.

**Figure 1:**
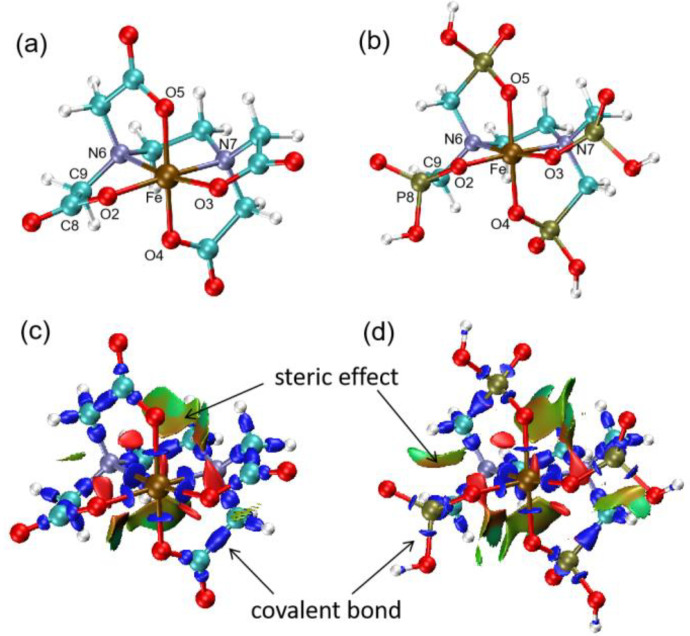
Optimized molecular structures and Interaction Region Indicator isosurfaces of Fe(EDTA)^−^ (**a**,**c**) and Fe(EDTMP)^−^ (**b**,**d**) complexes.

The EDTA and EDTMP molecules form 1:1 complexes with Fe^3+^ ions, as shown in Figure 1. The structure of the four carboxylic acid groups of EDTA and two nitrogen atoms coordinate with the Fe^3+^ ion in Fe(EDTA)^−^, forming a six-coordinated structure. The molecular structure of Fe(EDTMP)^−^ is similar to Fe(EDTA)^−^, except that the phosphonic acid group provides an oxygen atom for coordination. The optimized structures of the complexes are used to investigate their thermal decomposition pathways.

**Figure 2:**
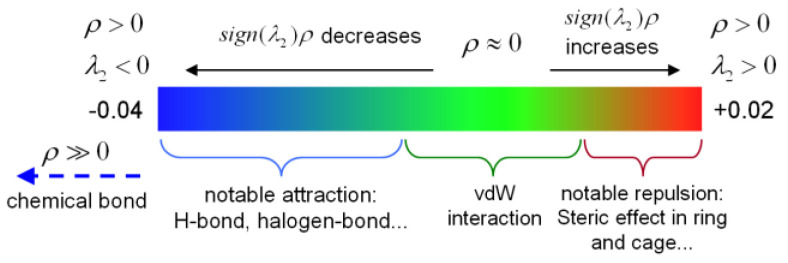
The color bar of the Interaction Region Indicator [36].

The Interaction Region Indicator (IRI) is a visualization method for studying weak interactions [36] which not only reflects chemical bonds but also displays weak interactions, such as van der Waals (vdW) forces, hydrogen bonds, electrostatic interactions, and steric hindrance [37,38]. The IRI isosurfaces of the two complexes are shown in Figure 1c,d. The color of the IRI isosurfaces (Figure 2) indicates the type of weak interactions. Green isosurfaces represent weak vdW interactions. If the color of the isosurface tends towards red, it indicates some steric hindrance, and if it is bright red, it suggests strong steric hindrance. A bluish isosurface represents significant attraction, such as hydrogen bonds. If the isosurface is entirely blue, it indicates either relatively strong weak interactions or chemical bond interactions.

It can be observed from Figure 1c,d that both complexes clearly display covalent bonds, intramolecular steric hindrance regions, intermolecular hydrogen bonds, and vdW interaction regions. There are red regions within the five-membered rings, indicating repulsive interactions. Weak attractive interactions, in green, are observed between carboxylic acid groups or between phosphonic acid groups. However, the interactions between coordinating groups in the two complexes are different. The brown region in Fe(EDTMP)^−^ is significantly larger than that in Fe(EDTA)^−^, indicating weak repulsive interactions between adjacent phosphonic acid groups in Fe(EDTMP)^−^. This is because the C atoms in the carboxylic acid groups of EDTA are of *sp^2^* hybridization, while the P atoms in the phosphonic acid groups of EDTMP are of *sp^3^* hybridization. The *sp^3^* hybridization has a larger spatial extension than the *sp^2^* hybridization, resulting in weak steric hindrance interactions between the phosphonic acid groups in Fe(EDTMP)^−^, and smaller steric hindrance interactions between the carboxylic acid groups in Fe(EDTA)^−^.

### 3.2. Tracking the Decomposition Trajectory of the Fe(EDTA)^−^ Complex

**Figure 3:**
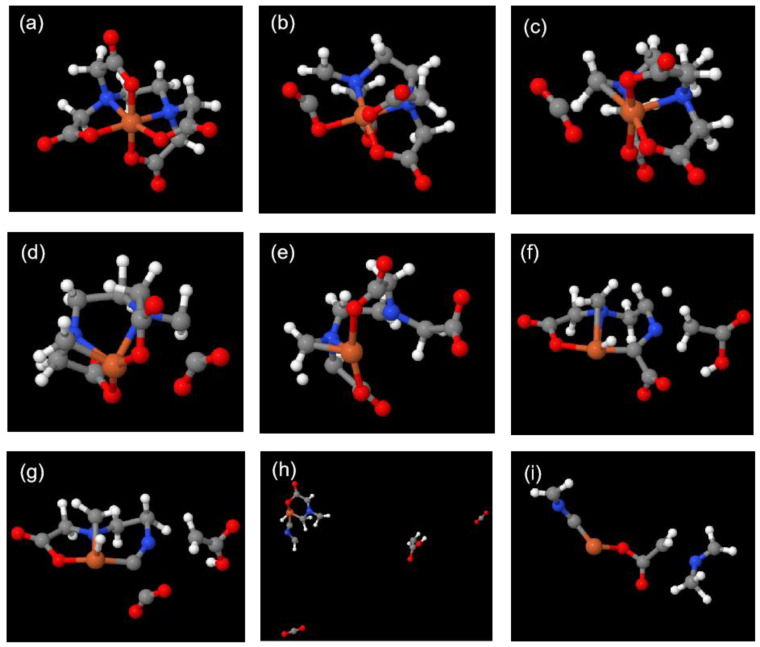
Snapshots of the decomposition trajectory of the Fe(EDTA)^−^ complex. ((**a**)—0 fs; (**b**)—1004 fs; (**c**)—1037 fs; (**d**)—2166 fs; (**e**)—2294 fs; (**f**)—3678 fs; (**g**)—3699 fs; (**h**)—4170 fs; (**i**)—4658 fs).

**Figure 4:**
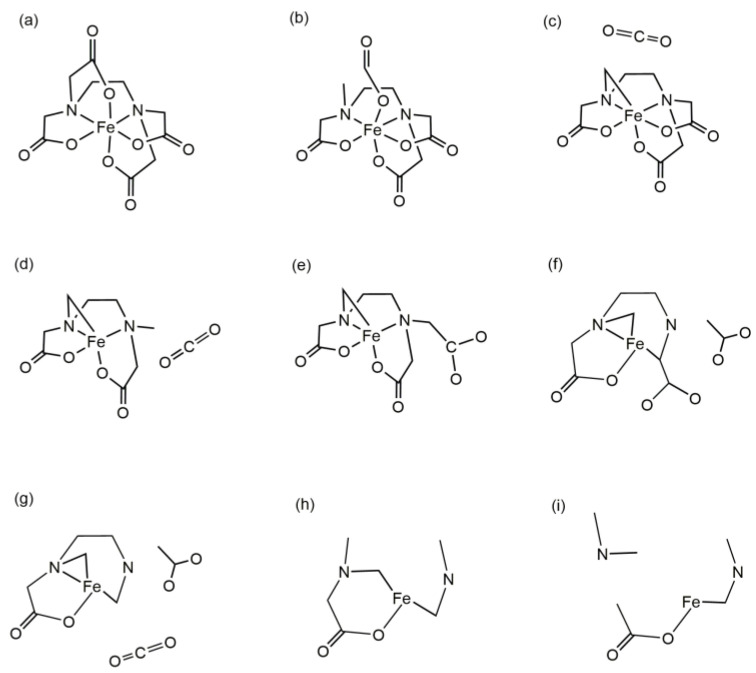
Scheme showing the decomposition reaction of the Fe(EDTA)^−^ complex (the figure order corresponds to that of Figure 3. (**a**)—0 fs; (**b**)—1004 fs; (**c**)—1037 fs; (**d**)—2166 fs; (**e**)—2294 fs; (**f**)—3678 fs; (**g**)—3699 fs; (**h**)—4170 fs; (**i**)—4658 fs).

The thermal decomposition process of the Fe(EDTA)^−^ complex was tracked to determine its stability at 3000 K. At 0 fs, Fe(EDTA)^−^ is a stable complex, with Fe and two N atoms forming a plane (Figure 3a and Figure 4a). Carboxyl groups parallel to this plane are denoted as parallel carboxyl groups, while those perpendicular to it are denoted as perpendicular carboxyl groups. From the molecular dynamics trajectory (Movie S1), it can be observed that Fe(EDTA)^−^ undergoes intense vibrations at high temperatures. At 1004 fs, a C-C bond of a perpendicular carboxyl group breaks, forming a weak connection between O=C=O and the Fe ion (Figure 3b and Figure 4b). At 1037 fs, O=C=O completely detaches from the Fe atom, forming a stable CO_2_ molecule, which leaves the complex (Figure 3c and Figure 4c). At 2166 fs, another parallel carboxyl group briefly forms CO_2_ but is quickly captured by the complex and does not leave (Figure 3d and Figure 4d). At 2294 fs, the Fe-N bond breaks (Figure 3e and Figure 4e). At 3678 fs, a carboxyl group methyl radical detaches from the complex (Figure 3f and Figure 4f). At 3699 fs, another CO_2_ molecule detaches from the complex (Figure 3g and Figure 4g). At 4170 fs, two CO_2_ molecules, one carboxyl group methyl radical, and the remaining complex form a cyclic molecule (Figure 3h and Figure 4h). At 4658 fs, the cyclic molecule breaks at the C-N bond, and the residual group coordinated with Fe also detaches from the Fe^3+^ ion, leading to the eventual decomposition of the complex (Figure 3i and Figure 4i). Ref. [17] mentions the generation of CO_2_ and intermediates in thermogravimetric analysis, which is consistent with our calculations.

### 3.3. Tracking the Decomposition Trajectory of the Fe(EDTMP)^−^ Complex

**Figure 5:**
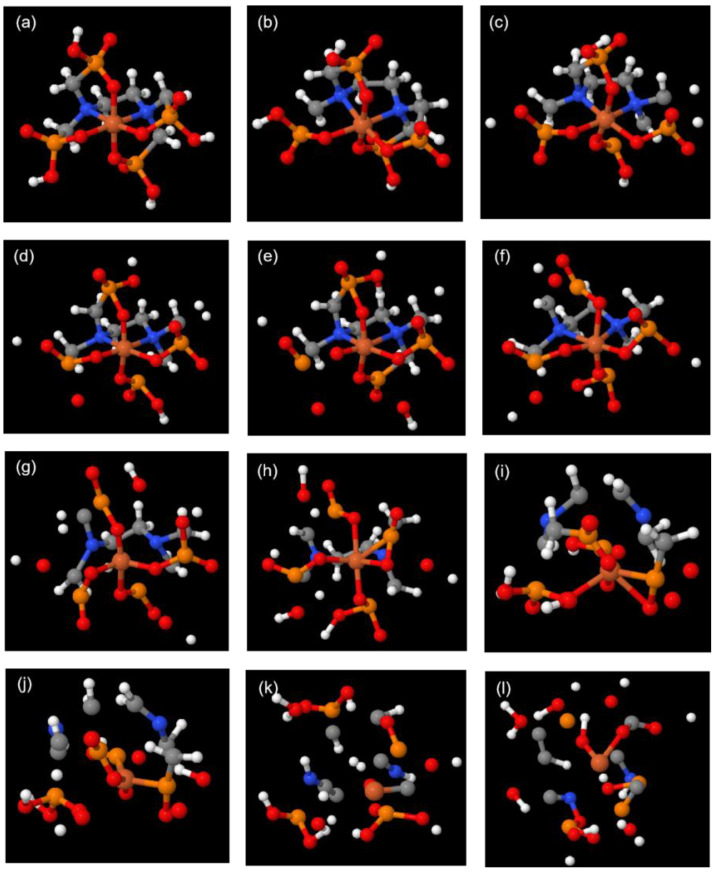
Snapshots of the decomposition trajectory of Fe(EDTMP)^−^ complex. ((**a**)—0 fs; (**b**)—2 fs; (**c**)—13 fs; (**d**)—15 fs; (**e**)—21 fs; (**f**)—39 fs; (**g**)—49 fs; (**h**)—61 fs; (**i**)—73 fs; (**j**)—80 fs; (**k**)—200 fs; (**l**)—400 fs).

**Figure 6:**
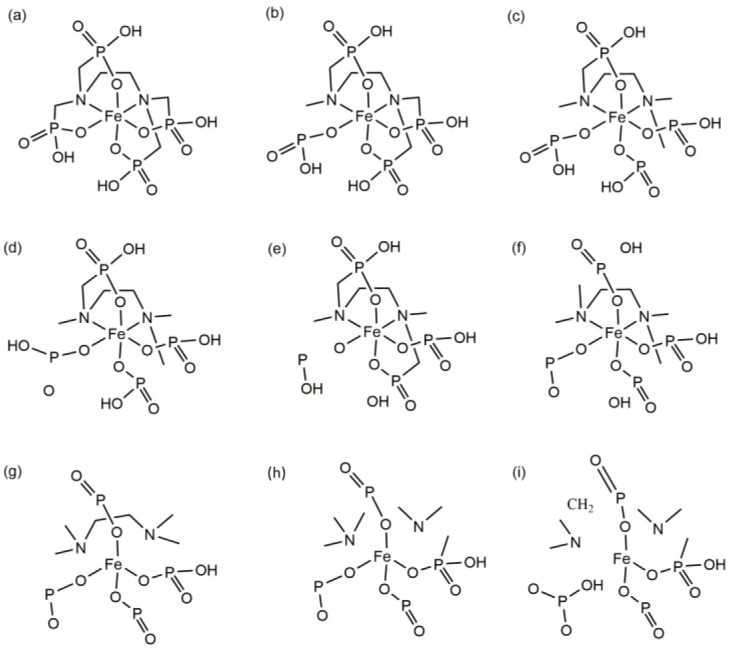
Scheme showing the decomposition reaction of the Fe(EDTMP)^−^ complex (the figure order corresponds to that of Figure 5. (**a**)—0 fs; (**b**)—2 fs; (**c**)—13 fs; (**d**)—15 fs; (**e**)—21 fs; (**f**)—39 fs; (**g**)—49 fs; (**h**)—73 fs; (**i**)—80 fs).

The Fe and two N atoms in Fe(EDTMP)^−^ are also considered to form a plane. Phosphonic acid groups parallel to this plane are denoted as parallel phosphonic acid groups, while those perpendicular to it are denoted as perpendicular phosphonic acid groups. Fe(EDTMP)^−^ undergoes intense vibrations at 3000 K, but unlike Fe(EDTA)^−^, it undergoes decomposition from the initial stages (Movie S2). At 2 fs, the P8-C9 bond breaks (Figure 5b and Figure 6b). At 13 fs, another perpendicular and a parallel P-C bond breaks (Figure 5c and Figure 6c). At 15 fs, an O atom from the parallel phosphonic acid group detaches, forming a free oxygen atom (Figure 5d and Figure 6d). At 21 fs, the P=O group from the parallel position detaches from the complex, and the OH group from the perpendicular phosphonic acid group also detaches (Figure 5e and Figure 6e). At 39 fs, the perpendicular P-C bond breaks, leaving all P-C bonds in the complex in a broken state (Figure 5f and Figure 6f). At 49 fs, both Fe-N bonds break, roughly dividing the complex into two parts: Fe with four incomplete phosphonic acid groups and (CH_2_)_2_-N-C-C-N-(CH_2_)_2_ (Figure 5g and Figure 6g). At 61 fs, the P-C bond below the perpendicular position dissociates through proton transfer (Figure 5h). At 73 fs, the C-C bond between (CH_2_)_2_-N-C-C-N-(CH_2_)_2_ breaks (Figure 5i and Figure 6h). At 80 fs, the N-C bond between C-N-(CH_2_)_2_ and the Fe-O bond break, leaving behind the H_2_PO_3_ moiety (Figure 5j and Figure 6i). By 200 fs, the complex has completely decomposed (Figure 5k).

### 3.4. Comparison of the Decomposition Reactions of the Fe(EDTA)^−^ and Fe(EDTMP)^−^ Complexes

From the decomposition processes of the Fe(EDTA)^−^ and Fe(EDTMP)^−^ complexes, it can be observed that, although their structures are similar, the differences in the carboxylic acid and phosphonic acid groups lead to completely different decomposition behaviors. The initial decomposition reactions of both complexes begin where the organic acid groups are connected to C, indicating that the C-C or P-C bonds play a decisive role in the decomposition reactions. However, the molecular structure of Fe(EDTA)^−^ is stable, and the C-C bonds start to break only after 1000 ps. By contrast, in the early stages of decomposition, the P-C bonds of Fe(EDTMP)^−^ break due to the presence of partial *π* bonds between C-C bonds, while P-C bonds do not have *π* bond components, making them more prone to breakage.

EDTA forms delocalized *π* bonds due to the presence of carboxylic acid groups, strengthening the coordination bond interactions of the complex, while the phosphonic acid groups in EDTMP only have *σ* bonds, making the covalent interactions of the coordination bonds in Fe(EDTA)^−^ stronger than those in Fe(EDTMP)^−^.

After the initial decomposition reaction, there are significant differences in the intermediate reaction pathways between the two complexes. Compared to Fe-O bonds, Fe-N bonds are less stable due to the lower negative charge carried by N atoms when compared to O atoms. This leads to the weaker coordination ability of Fe-N bonds when compared to Fe-O bonds, making them more prone to breakage. From these trajectories, it can be seen that Fe-N bonds break before Fe-O bonds. In the intermediate reaction process of Fe(EDTA)^−^, carboxylic acid groups easily form CO_2_, leading to the decomposition of the complex. In the intermediate reaction process of Fe(EDTMP)^−^, after the initial breakage of P-C bonds, the phosphonic acid groups themselves become unstable. Due to their larger volume compared to carboxylic acid groups, there is stronger repulsion between the phosphonic acid groups, leading to the decomposition of the phosphonic acid groups themselves.

## 4. Conclusions

This study investigated the molecular structures, decomposition trajectories, and decomposition processes of Fe(EDTA)^−^ and Fe(EDTMP)^−^ complexes. It was found that although their molecular structures are similar, the differences in the phosphonic acid and carboxylic acid groups make Fe(EDTMP)^−^ much easier to decompose compared to Fe(EDTA)^−^. The carboxylic acid groups in Fe(EDTA)^−^, due to the presence of delocalized *π* bonds, exhibit stable binding with Fe^3+^, not only stabilizing themselves but also the bond with the (CH_2_)_2_-N-C-C-N-(CH_2_)_2_ backbone. In contrast, the phosphonic acid groups in Fe(EDTMP)^−^ only have *σ* bonds between P and O, making them individually unstable and generally less stable when bonding with the (CH_2_)_2_-N-C-C-N-(CH_2_)_2_ backbone. Additionally, the repulsion between phosphonic acid groups is greater than that between carboxylic acid groups, leading to more intense vibrations at high temperatures and the easier decomposition of Fe(EDTMP)^−^. The structural characteristics of Fe(EDTMP)^−^ make it more readily degradable in the environment.

These findings offer insights for designing organic acid molecules with chelating properties and suggest that incorporating phosphonic acid groups can facilitate easier degradation after forming chelates with metal ions due to the ease of breaking P-C bonds. This study provides a basis for research on the degradation pathways of organic compounds in industrial wastewater, such as organic acid industrial cleaners, circulating cooling water scale and corrosion inhibitors, and chelating agents.

## Figures and Tables

**Table 1:** Geometry parameters of the two complexes.

Fe(EDTA)^−^		Fe(EDTMP)^−^	
Bond	Dist/nm	Bond	Dist/nm
Fe–O2	0.1968	Fe–O2	0.1979
Fe–O3	0.1968	Fe–O3	0.1979
Fe–O4	0.1924	Fe–O4	0.1926
Fe–O5	0.1924	Fe–O5	0.1926
Fe–N6	0.2003	Fe–N6	0.2057
Fe–N7	0.2003	Fe–N7	0.2059
O2–C8	0.1290	O2–P8	0.1537
C8–C9	0.1533	C9–P8	0.1845
C9–N6	0.1481	C9–N6	0.1489

## Data Availability

All the data supporting the findings of this study are available within the article and from the corresponding author upon request.

## Supplementary Materials

The following supporting information can be downloaded at: https://www.mdpi.com/article/10.3390/molecules29184362/s1, Movie S1 and Movie S2.
